# Synchronous triple cancers of synchronous endometrial and ovarian carcinoma complicated by renal cancer: a case report

**DOI:** 10.3389/fonc.2026.1668256

**Published:** 2026-03-11

**Authors:** Mu Xu, Jianhui Fu, Liangzhi Cai

**Affiliations:** 1 Department of Gynecology, Fujian Maternity and Child Health Hospital, College of Clinical Medicine for Obstetrics & Gynecology and Pediatrics, Fujian Medical University, Fuzhou, China; 2 College of Clinical Medicine for Obstetrics & Gynecology and Pediatrics, Fujian Medical University, Fuzhou, China; 3 Department of Pathology, Fujian Maternity and Child Health Hospital, College of Clinical Medicine for Obstetrics & Gynecology and Pediatrics, Fujian Medical University, Fuzhou, China

**Keywords:** case report, endometrial cancer, multiple primary cancers, ovarian cancer, renal cancer

## Abstract

**Background:**

Synchronous primary carcinoma of the endometrium and ovary is rare in clinical practice. Distinguishing between synchronous primary carcinomas and metastatic carcinomas is very important for the selection of subsequent treatment regimens. In addition, triple primary malignancies are even rarer. Here, we report a case of synchronous triple primary malignancies, including primary carcinomas of the endometrium, ovary, and kidney.

**Case presentation:**

We report a 60-year-old female patient who was diagnosed as synchronous primary carcinoma of the endometrium and ovary. Comprehensive staging surgery were performed. The molecular subtyping of endometrial cancer was mismatch repair deficiency. The whole-abdomen MRI was performed before the radiotherapy and a mass was found in the right renal parenchyma. The pathology showed papillary renal cell carcinoma. The result of germline genetic testing was negative (also negative for MLH1、MSH2、MSH6、PMS2、EPCAM). No recurrence or metastasis was observed during a 12-month follow-up period.

**Conclusion:**

This case report adds to the limited literature on synchronous triple primary malignancies. For synchronous endometrial and ovarian cancer, distinguishing between primary tumors and metastases is clinically critical. Notably, the presence of MMRd subtype in such synchronous tumors does not inherently indicate Lynch syndrome. This case underscores the importance of integrating molecular testing and clinical manifestations to accurately diagnose multiple primary malignancies and formulate tailored treatment plans.

## Introduction

The probability of two or more primary malignancies occur simultaneously in the female reproductive system is 1% - 2% (1). Among these simultaneous primary malignancies, synchronous primary endometrial and ovarian cancer (SEOC) is the most common, accounting for 50% - 70% (1). Research shows that SEOC accounts for approximately 5% of endometrial cancers and 2.6% - 10% of ovarian cancers (2). SEOC occurs at a relatively younger median age of 46.6 - 48.3 years, younger than that of endometrial cancer or ovarian cancer (2). Among SEOC patients, pre-menopausal patients are the majority, accounting for 68% (3, 4). Clinically, primary double cancers are often misdiagnosed as metastatic cancers, which challenge clinicians and pathologists. In contrast to cases of ovarian cancer with endometrial metastasis (OEC) or endometrial cancer with ovarian metastasis (ECO), SEOC is at an earlier tumor stage and has a lower pathological grade at the time of onset, and thus has a better prognosis (5–8). Therefore, distinguishing between double primary cancers and metastatic cancers has a significant impact on the choice of treatment methods and prognosis. Differentiation should be carried out to achieve timely and accurate diagnosis, so as to avoid over-treatment or under-treatment. Primary triple cancers with SEOC combined with other cancers are even rarer in clinical practice and significantly affect the treatment and prognosis of patients. This paper analyzes a case of primary triple cancers with SEOC combined with renal cancer, which haven’t been reported before, explores its clinical and pathological characteristics as well as the diagnosis and treatment process, aiming to provide reference for clinical diagnosis and treatment decisions of primary triple cancers.

## Case presentation

A 60 -year-old postmenopausal woman (gravida 2, para 1, no history of abortion) who underwent natural menopause at 55 years old came to our hospital with a 9 - month history of vaginal bleeding without associated abdominal pain. An outside hospital’s transvaginal color ultrasound showed a 2.4 - cm thickened endometrium and a 9.1×7.0 - cm cystic - solid mass in the right adnexal area. Segmented curettage was performed and pathology showed endometrial endometrioid adenocarcinoma (Grade 2). The patient was treated for hypertension for the past 2 years. She had no history of diabetes mellitus and reported that she did not use tobacco or alcohol. She had no history of exposure to oral estrogen, and her family history was unremarkable.

### Laboratory and imaging examinations

**Figure 1:**
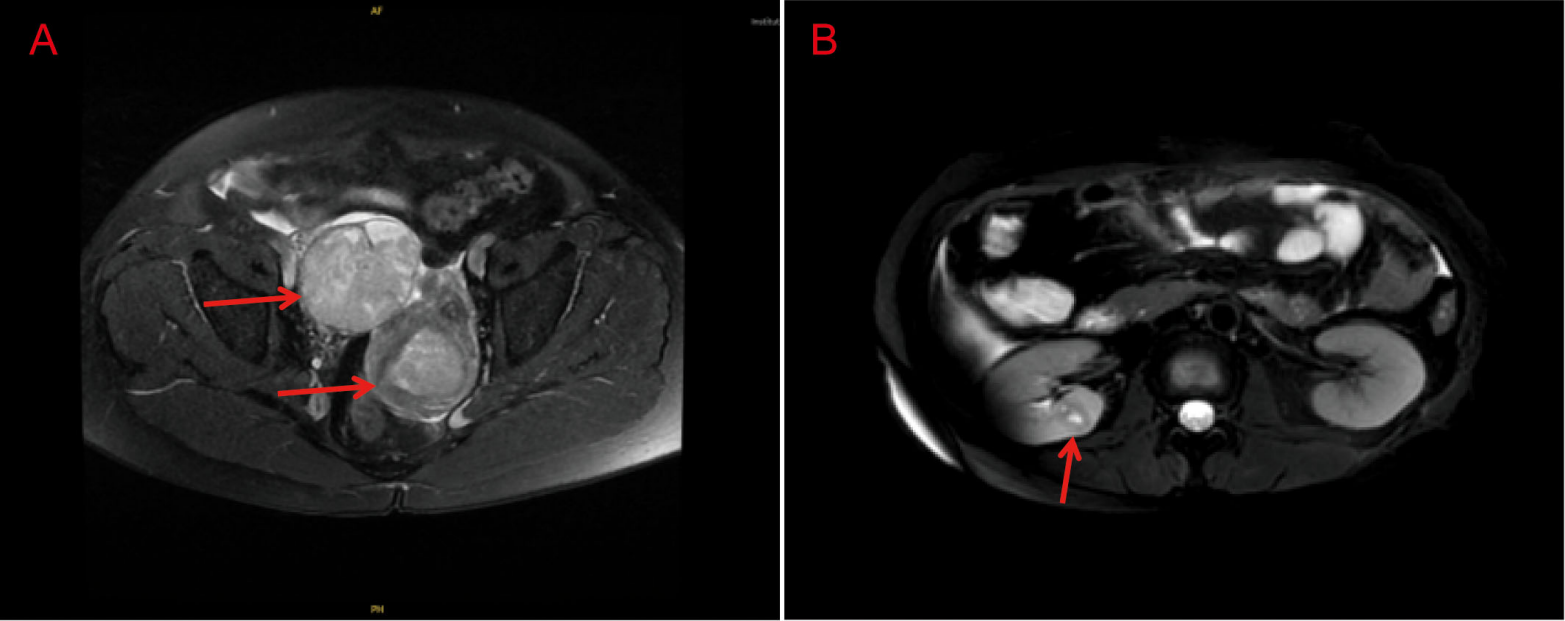
Imaging findings of the patient. **(A)** MRI scan showed uterine cavity mass (4.3×3.0×3.7cm) with T2 hyperintensity and heterogeneous enhancement; right adnexal irregular solid mass (8.2×6.4×6.5cm) with cystic components and peripheral enhancement, no pelvic lymphadenopathy or peritoneal nodules. **(B)** Right renal parenchymal mass with clear borders, T1 hypointensity, T2 isointensity, and mild heterogeneous enhancement.

Preoperative serum follicle-stimulating hormone (FSH) was 89.3 mIU/ml, luteinizing hormone (LH) was 31.2 mIU/ml, estradiol (E2) was 18.5 pg/ml. Serum carbohydrate antigen (Ca) - 125 was 344.6U/mL; Ca199 was 2157.62U/ml; HE4 was 303.03pmol/L; ROMA was 82.76. Carcinoembryonic antigen (CEA), Ca153, alpha fetoprotein (AFP) and squamous cell carcinoma antigen (SCC) were in the normal range. Human papillomavirus (HPV) DNA testing was negative. Preoperative pelvic magnetic resonance imaging (MRI) revealed a uterine cavity mass (size about 4.3cm×3.0cm×3.7cm) with heterogeneous enhancement, infiltrating the superficial myometrium (<1/2 myometrial thickness). No abnormal signal in the cervical stroma. Pelvic and para-aortic lymph nodes were not enlarged (short-axis diameter <1cm). Right adnexal showed irregular solid mass (size about 8.2×6.4×6.5cm) with cystic components and peripheral enhancement, no peritoneal nodules or ascites (**Figure 1A**). Both the gastroscopy and colonoscopy results are normal, and the result of the whole - abdomen color ultrasound was also normal. Compared to CT, MRI had superior soft-tissue resolution for pelvic organs, which was recommended as the first-line imaging for endometrial/ovarian cancer staging. The patient had no evidence of distant metastasis on MRI and gastrointestinal endoscopy. Then CT and PET-CT were not performed.

### Treatment and pathology

**Figure 2:**
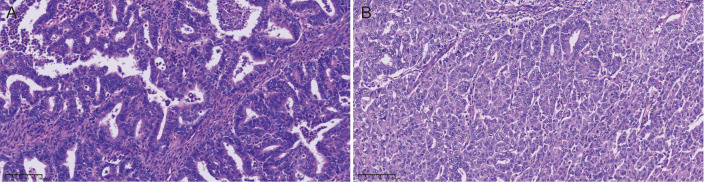
Microscopic evaluation. **(A)** Ovarian adenocarcinoma with endometrioid differentiation (H&E, ×200) with MSH2(-)/MSH6(-). **(B)** Poorly differentiated endometrioid endometrial adenocarcinoma (H&E, ×200) with MLH1(-)/PMS2(-).

Transabdominal extrafascial hysterectomy and bilateral oophorosalpingectomy were performed. During intraoperative exploration, no abnormality was found in the abdominal pelvic cavity. No obvious enlarged pelvic lymph nodes were observed. The uterus was enlarged to 7.0cm×5.5cm×4.2cm, measured intraoperatively with a sterile ruler. A cystic lesion with a solid tumor in the right ovary was found. The tumor was cystic-solid with an intact capsule (no rupture), measuring 9.0cm×7.5cm×6.8cm. The solid component was intracystic (not exophytic), with a tan cut surface and no necrosis. The bilateral fallopian tubes and the left ovary were unremarkable. The Douglas fossa was closed. Frozen section findings confirmed endometrial carcinoma with <50% myometrial invasion (no internal cervical os involvement) and right adnexal adenocarcinoma (subtype undetermined pending final pathology). Given the high suspicion of ovarian malignancy, pelvic and para-aortic lymph node dissection plus omentectomy were conducted for surgical staging (9). Histopathological examination showed endometrioid carcinoma (G3) which infiltrated the superficial half of the myometrium, without lymphovascular invasion or cervical stroma invasion. On immunohistochemistry, tumor cells were positive for estrogen receptor (ER, 40%+), progesterone receptor (PR, 40%+), MSH2 and MSH6, but negative for MLH1 and PMS2. Ki-67 index was 80%. Histopathological examination of the right adnexa showed endometrioid carcinoma of the right fallopian tube - ovary (G2) without lymphovascular invasion or invasion of the fallopian tube myometrium (**Figure 2**). Tumor cells were positive for estrogen receptor (ER, 10%+), progesterone receptor (PR, 10%+), MLH1 and PMS2, but negative for MSH2 and MSH6 on immunohistochemistry. Ki-67 index was 60%. Metastasis was not detected in lymph nodes. Tumor cells were negative in the peritoneal washing liquid. The molecular subtyping of endometrial cancer was mismatch repair deficiency (MMRd).

Following multidisciplinary discussion, a diagnosis of synchronous primary double cancers was established. For the endometrial carcinoma, given the risk factors including grade 3 histology, advanced age of 60 years, and superficial myometrial invasion, adjuvant radiotherapy after surgery is recommended in accordance with current clinical guidelines (10). Adjuvant chemotherapy is recommended as the subsequent treatment for the ovarian carcinoma (9). One month after surgery, a whole-abdominal MRI scan was performed prior to the radiotherapy. A mass was found in the right renal parenchyma with clear boundary and mild heterogeneous enhancement (approximately 1.4×1.3×1.3 cm in size) (**Figure 1B**). However, the preoperative abdomen ultrasound showed no abnormality. Laparoscopic right partial nephrectomy was performed two months after the initial gynecological surgery. The pathology showed papillary renal cell carcinoma (International Society of Urological Pathology (ISUP) grade 1) without lymphatic or vascular invasion. There was no invasion of the perirenal adipose tissue and lymph nodes. Tumor cells were positive for FH, SDHB, PAX8, CK7, P504S, but negative for CD117, 2SC, CK20 and TFE3 on immunohistochemistry (The distinguishing features between the three tumors were summarized in **Table 1**). Regular follow-up review is required. The germline genetic testing was performed and the result was negative (also negative for MLH1、MSH2、MSH6、PMS2、EPCAM).

**Table 1:** The distinguishing features between the three tumors were summarized in **Table 1**.

Distinguishing dimensions	Endometrial tumor	Ovarian tumor	Renal tumor	Key rationale for synchronous primaries (vs. metastasis)
Histological Type	Endometrioid adenocarcinoma	Endometrioid adenocarcinoma	Papillary renal cell carcinoma	Distinct tissue origins (endometrium vs. ovary vs. renal tubule epithelium) rule out single primary with metastasis
Pathological Grade	G3	G2	ISUP Grade 1	Metastatic tumors typically retain the primary’s grade; discordant grades support independent carcinogenesis
MMR Protein Expression	MLH1(-), PMS2(-), MSH2 (+), MSH6(+) (MMRd)	MLH1(+), PMS2(+), MSH2(-), MSH6(-) (pMMR)	/	Discordant MMR profiles (endometrial MMRd vs. Ovarian pMMR) exclude clonal relatedness (metastatic tumors share primary MMR phenotype)
ER/PR/Ki-67 (IHC)	ER(40%+), PR(40%+), Ki-67 (80%)	ER(10%+), PR(10%+), Ki-67 (60%)	ER(-), PR(-), PAX8(+), P504S(+)	Variable ER/PR expression (gynecologic tumors) and lineage-specific markers (PAX8、P504S for RCC) confirm distinct tumor biology
Invasion Depth/Range	Superficial myometrial invasion (<1/2), confined to uterus	No fallopian tube myometrial invasion, confined to ovary	No perirenal adipose/vascular invasion, confined to renal parenchyma	No deep invasion, lymphovascular invasion (LVI), or extrapelvic spread showed non-metastatic tumors
Lymphovascul-ar Invasion	Negative	Negative	Negative	LVI is a key marker of metastatic potential; absence supports primary tumors without dissemination
Tumor Origin	Endometrial glandular epithelium	Ovarian surface epithelium/endometriotic foci	Renal tubular epithelial cells	Histogenetically distinct origins (gynecologic vs. urinary system) exclude metastasis between systems
Imaging Characteristics	Uterine cavity mass (4.3×3.0×3.7cm), heterogeneous enhancement	Right adnexal cystic-solid mass (8.2×6.4×6.5cm), peripheral enhancement	Right renal parenchymal mass (1.4×1.3×1.3cm), clear boundary, mild heterogeneous enhancement	Anatomically isolated tumors with no peritoneal nodules, lymphadenopathy, or distant metastasis—consistent with synchronous primaries
Gross Pathological Features	Intracavitary mass	Cystic-solid with intact capsule, intracystic solid component	Well-circumscribed, tan cut surface, no necrosis	Intact ovarian capsule and lack of exophytic growth rule out ovarian metastasis; well-circumscribed RCC is typical of primary disease

The patient underwent germline genetic testing (OncoDeficiency^®^ 36-gene panel). Genomic DNA was extracted from the patient’s peripheral blood (EDTA anticoagulation) using standard phenol-chloroform extraction, with quantification via Qubit 4.0 (DNA yield: 4224 ng, meeting the ≥200 ng requirement) and degradation assessment via agarose gel electrophoresis (main band ≥10 kb, no significant degradation).Targeted exon capture-based next-generation sequencing (NGS) was performed on the MGISEQ2000/NextSeq550 platform. The capture panel covered the entire exonic regions and flanking splice sites (± 15–20 bp) of 36 genes (including MLH1, MSH2, MSH6, PMS2, EPCAM).

### Outcome and follow-up

Based on the multidisciplinary team (MDT) discussion, the final diagnoses were as follows: endometrioid adenocarcinoma, FIGO stage IIC (G3, 2023); ovarian endometrioid adenocarcinoma, FIGO stage IIA (G2, 2014); and papillary renal cell carcinoma of the right kidney (ISUP grade 1). After surgery, the patient received radiotherapy (pelvic external beam radiation therapy (50.4 Gy in 28 fractions, 1.8 Gy per fraction, 5 fractions per week) and six courses of carboplatin plus paclitaxel chemotherapy (Carboplatin (AUC 5) + paclitaxel (170 mg/m²) every 3 weeks, initiated concurrently with radiotherapy) for gynecological oncology. According to the EAU Guidelines (11), no adjuvant therapy was indicated for papillary renal cell carcinoma, and only regular surveillance was required. At the time of this writing, 1 year has passed since the operation. So far, there has been no evidence of recurrence. The MRI and tumor markers are normal. The clinical timeline was showed in **Table 2**.

**Table 2:** The clinical timeline in diagnosis.

Date	Event
Month 1	Patient presents with 9-month vaginal bleeding; external transvaginal ultrasound + segmented curettage (endometrial endometrioid adenocarcinoma G2).
Month 1 + 1w	Admission to our hospital; serum tumor markers + pelvic MRI + gastroscopy/colonoscopy (confirm gynecologic masses, rule out gastrointestinal primaries).
Month 1 + 2w	Transabdominal hysterectomy + bilateral salpingo-oophorectomy + lymphadenectomy + omentectomy.
Month 1 + 3w	Postoperative pathology confirms SEOC (endometrial G3; ovarian G2). The molecular subtyping showed MMRd.
Month 2 + 1w	Pre-radiotherapy whole-abdomen MRI detects right renal mass (1.4×1.3×1.3cm).
Month 2 + 2w	Laparoscopic right partial nephrectomy (pathology: papillary RCC ISUP grade 1). genetic testing initiated
Month 3-8	Adjuvant radiotherapy (50.4 Gy/28 fractions) + 6 cycles of carboplatin + paclitaxel.
Month 14	12-month follow-up: Normal tumor markers + MRI, no recurrence.

## Discussion

Differentiating between primary double cancers and the presence of metastasis is crucial. If the staging is uncertain, it will directly affect the patient’s treatment strategy and also have different impacts on the prognosis. The 2023 staging system newly updated by the International Federation of Gynecology and Obstetrics (FIGO) has introduced a new subcategory, Stage IA3, which is specifically for SEOC (12). In these patients, reducing the intensity of adjuvant therapy is recommended. Matsuo (13) revealed that patients with stage IA3 disease generally have an overall favorable prognosis, regardless of whether they receive adjuvant therapy or not. Nevertheless, if a patient’s condition fails to meet the criteria for Stage IA3, they should be classified as at least Stage IIIA1. In such cases, adjuvant therapy is recommended to enhance the survival rate.

If the pathology of the ovarian and endometrial lesions is different, it can be judged as primary double cancers. If they are of the same pathological type, such as both being endometrioid adenocarcinoma, it is often difficult to differentiate. The pathological criteria were first proposed by Ulbright and Roth (14) to differentiate between metastatic cancer and independent double primary cancers. The diagnosis of metastatic cancer was based on the following criteria: small ovary (<5cm), bilateral ovarian involvement with multinodular ovaries, deep myometrial invasion of the uterus, vascular invasion, and the fallopian tube involvement. As the research advanced, Scully (15) modified and proposed the clinical-pathological diagnostic criteria in 1998. However, conventional pathological diagnosis has certain limitations and is vulnerable to subjective factors and experience.

Currently, some studies have conducted differential diagnosis by detecting molecular characteristics. Through targeted and exome sequencing, Anglesio (16) found that among 11 SEOC patients meeting the pathological diagnostic criteria, 10 cases had a clonal connection between uterine and ovarian cancer lesions. It is speculated that these tumor cells may not have undergone apoptosis. They detached from the primary tumor, metastasized to the ovarian tissue, and were restricted by the ovarian microenvironment, preventing further metastasis. This phenomenon of restricted metastasis is called “microenvironment restriction” and exhibits indolent characteristics. Another study also showed that 92% (46/50) of SEOCs had clonal correlation, and their molecular profiles were extremely similar to those of endometrial cancer in The Cancer Genome Atlas (TCGA) (17). Therefore, it is believed that these SEOCs are actually metastatic endometrial cancers. However, this study failed to explain why the prognosis of these SEOC patients is better than that of metastatic tumors. In addition, diagnosis can also be carried out through microsatellite instability (MSI), loss of heterozygosity, immunohistochemistry and gene mutation screening. Research shows (18) that the probability of MSI occurring in SEOC is twice that in primary single cancer, accompanied by a high frequency of PTEN gene and β-catenin gene mutations. However, molecular research also has its limitations. Research shows that most SEOCs are clonally related. Considering the possible intratumoral heterogeneity, these studies may not be able to precisely determine the origin of the clones and the direction of tumor metastasis (19–21). Moreover, these studies have not elucidated the mechanism of “restricted metastasis” in such tumors, nor can they explain why the prognosis of these SEOC patients is better than that of patients with single cancer metastasis. Therefore, it is still necessary to combine pathological examinations and make a comprehensive judgment based on both in clinical practice.

In this study, the patient’s endometrial lesion only infiltrates the superficial myometrium, involving less than 1/2 of the myometrial thickness, without lymphovascular invasion, and the lesions are limited to the endometrium and ovaries. Regarding pathological grading, the endometrial cancer was G3, and the adnexal tumor was G2. Immunohistochemically, in the endometrium, the molecular subtype is MMRd, with MSH2(+), MSH6(+), MLH1 (–), and PMS2(-). In the adnexal tumor, MLH1 and PMS2 were positive. The discordant MMR IHC patterns are a key feature supporting independent primaries. In metastatic disease, MMR protein expression is typically consistent with the primary tumor (21). This discordance suggests distinct molecular pathogenesis: the endometrial tumor developed MMRd via MLH1/PMS2 loss (sporadic, no methylation), while the ovarian tumor exhibits MSH2/MSH6 loss (also sporadic, as germline testing was negative). This pattern has been reported in 15% of SEOC cases (20) and further confirms the synchronous primary nature of the gynecologic tumors. Considering the differences from these aspects, it is considered synchronous primary endometrial and ovarian cancer.

The routine whole-abdomen MRI examination before radiotherapy suggested a right kidney mass, with a high possibility of malignant tumor. Further surgical treatment was carried out, and the postoperative pathology confirmed it as papillary renal cell carcinoma (ISUP 1). Histologically, the right renal cell carcinoma originated from renal tubular epithelial cells, which was different from the origin of gynecological endometrioid carcinoma (detailed in **Table 2**). Therefore, it is considered to be multiple primary cancers. When a patient has two or more malignant tumors simultaneously, it is called multiple primary malignancies (MPMN), which was first defined by Warren and Gates (22). If the occurrence time is within 6 months, it is called synchronous cancer, and if it exceeds 6 months, it is called metachronous cancer. The incidence of synchronous cancer is lower than that of metachronous cancer. The incidence of MPMN is relatively low. According to the data of the International Association of Cancer Registries and the International Agency for Research on Cancer (IACR/IARC), the overall incidence of MPMN is 2.4%-17% (23). Clinically, double primary cancers are more common, while triple or more primary cancers are relatively rare. Patients with a genetic background of malignant tumors may be susceptible to MPMN.

The most common sites of MPMN are the digestive system, breast, and respiratory system. After inquiring about the patient’s family history, it was found that there was no hereditary history of malignant tumors among family members. In recent years, some studies have found that 19% of Lynch syndrome - related ovarian cancer patients may have synchronous endometrial cancer (24); among Lynch syndrome - related endometrial cancer patients, the incidence of SEOC is as high as 22% (25). Lynch syndrome is an autosomal dominant genetic disease caused by mutations in four mismatch repair genes (MLH1, MSH2, MSH6, and PMS2). Its main feature is susceptibility to multi-system malignant tumors, such as colorectal, gastric, endometrial, ovarian, liver, kidney and skin cancers. This patient had SEOC, and the molecular typing indicated MMRd. The patient was also complicated with kidney cancer, so there was a possibility of Lynch syndrome. Therefore, we further conducted germline genetic testing, and the result was negative.

MLH1 promoter methylation is a useful indicator for differentiating between sporadic and hereditary tumors, and it also helps in the diagnosis of Lynch syndrome and the assessment of prognosis (26). The patient underwent germline genetic testing using peripheral blood, which focuses on inherited genetic variants rather than somatic epigenetic alterations in tumor tissues. MLH1 promoter methylation is a somatic event typically detected via tumor tissue analysis, which was not feasible here due to the unavailability of reserved tumor tissue samples. MLH1 methylation is primarily used to distinguish sporadic mismatch repair deficiency from hereditary dMMR (Lynch syndrome)—sporadic dMMR often involves MLH1 methylation, while Lynch syndrome is driven by germline MMR gene mutations. However, in this patient, both germline MMR gene testing (MLH1, MSH2, MSH6, PMS2, EPCAM) and tumor dMMR screening were negative, meaning methylation testing would not alter the current conclusion. MLH1 methylation testing could further confirm sporadic etiology if tumor tissue is available.

Due to the low incidence of SEOC, there is no clear treatment guideline. The main treatment plan, involving surgery, radiotherapy, chemotherapy, targeted therapy and immunotherapy, needs to be comprehensively considered. The treatment principle is the same as that of single gynecological malignant tumors and should be considered according to tumor stage and pathological type (5, 10, 27). A comprehensive treatment plan including surgery and adjuvant radiotherapy and chemotherapy is adopted. Chemotherapy, as a systemic treatment, plays a crucial role and can improve the long - term survival rate and quality of life of patients. The first - line regimen is mainly the paclitaxel and platinum drug (TC) regimen. According to the patient’s pathological results, the renal cell carcinoma is in the early stage and requires regular observation and follow-up. Both endometrial cancer and ovarian cancer have intermediate - high - risk factors (for endometrial cancer: 60 years old, G3; for ovarian cancer: stage II, G2). According to the guidelines (9, 10), radiotherapy and chemotherapy are administered. Since it was not a metastatic cancer, immunotherapy was not currently being considered. At present, the patient has completed the treatment, the tumor markers have dropped to normal levels, and no recurrent lesions have been detected in imaging examinations. The patient is under further follow-up treatment.

In summary, this case report describes a rare synchronous triple primary cancer involving endometrial cancer, ovarian cancer, and renal cancer. For synchronous endometrial and ovarian cancers, it is crucial to distinguish between synchronous primary double cancer and metastatic cancer, as this has significant implications for the selection of subsequent treatment regimens and the prediction of prognosis for patients. Additionally, triple primary cancers are even rarer clinically. If it is SEOC combined with other tumors, especially when the molecular subtype is MMRd, it is necessary to differentiate from Lynch syndrome, and genetic testing can be carried out for assistance in diagnosis when necessary. What’s more, whole-abdominal MRI/CT is recommended to evaluate extra-pelvic sites for high-risk SEOC. Meanwhile, formulating an individualized comprehensive treatment plan can contribute to improving the survival rate and quality of life of patients.

## Data Availability

The data supporting the conclusions of this article is available from corresponding author on reasonable request.
